# Prevention in Dentistry To-Day

**Published:** 1912-10-15

**Authors:** J. Morgan Howe

**Affiliations:** New York


### PREVENTION IN DENTISTRY TO-DAY.

BY DR. J. MORGAN HOWE, NEW YORK.

Signs are multiplying that there is a growing appreciation of the necessity for preventive measures being discovered and put into active operation. The larger views we have been able to take in recent years have brought a realization that if civilized mankind is to be rescued from physical decadence along dental lines, prevention must be the means, for it can easily be seen that there can never be enough dentists to treat and repair damaged teeth for all that need it. Only the favored few can be thus helped, whether they are those who can themselves pay for the service, or those who must be paid for by others, by private benevolence, or from the public funds, or by the generous gratuity of the dentists themselves.

The revelations made by the systematic examinations of children's teeth, in the experiences of free dental clinics, and the activities of dental hygiene propaganda, have all tended to awaken added interest in study of prevention, which appears in an increased number of published communications on subjects germain to the etiology of dental disease or its prevention.

We have also the continued interest and support given to the investigations begun by the New York Institute of Stomatology three years ago, under the direction generously given without remuneration by Prof. William J. Gies, Ph.D., who is to make a report of this last year's study of the possible causes of decay at the October meeting of the First District Dental Society of New York. Interest has centered on caries, no doubt because of the greater loss occasioned by it, and of its greater effect on the young.

Special attention may well be directed to two books that have been published this year in London, both having the same title, *The Prevention of Dental Caries*, and both making the claim to having discovered effectual preventive measures. This is rather startling, and as we recognize the urgency of demand for just such information as these two books claim to furnish, further consideration of their contents should be given them than has been accorded by the reviews published in our periodicals. Dr. J. Sim Wallace in *The Prevention of Dental Caries* begins his Introduction[1] with the statement that "Dental Caries is one of the most easily and certainly preventable of diseases, and there would seem now to be no valid excuse for the bringing up of children with decayed teeth." He seems to fear that commercial and other considerations will resist the "diffusion of the truth," but he says, nevertheless, that the profession has passed from the "most doleful pessimism" to "a most optimistic attitude," and "instead of anticipating the continued increase of dental caries, we confidently anticipate its more or less complete extinction."

The author does not believe that susceptibility resides in the physical character of the teeth, and attaches little importance to qualities of saliva and mucus, for "we are able by altering the dietetic *regime* to make anyone practically immune to caries." He says "*the cause of caries is the prolonged retention or stagnation of fermentable carbohydrates in more or less immediate contact with the teeth and undisturbed by the free access of saliva.*" The dietetic regime that is claimed to effect practical immunity is required to have a physical consistency that is stimulating to pleasurable mastication, and is calculated "to secure physiological cleanliness of the mouth," and by other qualities such as acidity, to be stimulating to "saliva rich in ptyalin."

"We may divide foodstuffs," he says, "into two classes—namely, those which tend to leave viscous and fermentable carbohydrates about the teeth and those which tend to brush them away," and there is appended a list of the two

1The Prevention of Dental Caries, by J. Sim Wallace, D.Sc., M.D., L.D.S. THE DENTAL RECORD, London, 1912.

classes of food, those "not cleansing and liable to induce dental caries," those that are "farinaceous and sugary food in general without fibrous elements;" and, on the other hand, food that is "cleansing and preventive of dental caries. Fibrous foods generally."

Dr. Wallace does not regard the toothbrush as of much help in preventing caries. He says: "What statistics we have seem to show but little reliance can be put on artificial procedures. Thus in the better class schools, where the toothbrush is at least sometimes used, there is no evidence that the teeth are better than in schools where the brush is not used;" and, further, "the cleansing power of true or effectual mastication is better almost beyond comparison than artificial cleaning."

These views on the effects of certain kinds of food and of the influence of acid food, are given further emphasis when we find in the other publication to which our attention is called, an amplification of similar dietetic rules, based on independent research work, with quite as confident claims of prevention of caries.

This appears to be the most comprehensive and systematic work on causes and prevention since Miller's classical work. Dr. Pickerill says in his Introduction:[1] "*The author is convinced that, by means and methods subsequently to be described, not only may the occurrence of caries be prevented, but in some cases actually 'cured.'*

"*In several patients (children) who have been under constant observation during the past two years, and in whom caries was commencing, not only have no more carious cavities appeared, but those that were present have not progressed, and the surface has become quite hard.*"

The author reviews the history of attempts at prevention, and he also gives the percentage of occurrence of caries in the teeth of different races of men among savage and uncivilized, as well as the civilized races of to-day. The

---

[1]The Prevention of Dental Caries and Oral Sepsis, by H. P. Pickerill, M. D., Ph.B., M.D.S. (Birm.), L.D.S., Eng., Prof. of Dentistry and Director of the Dental School in the University of Otago, N. Z. London, Bailliere, Tindall and Cox, 1912.

pathology of the disease is described, and the various resistance of teeth of different individuals and races as indicated by the variations in the minute ridges or "imbrication lines" discoverable on the surface of enamel. Differences are noted that exist in the hardness of enamel as shown by the geological method of scratching, as the relative hardness of stones and gems is determined, and also the density and relative permeability of the structure. Then the saliva and mucus, with their variations in quantity and quality as affected by different foodstuffs and by dentifrices and antiseptics, are all tested by original methods. The means of prevention claimed by Dr. Pickerill to be effectual is purely dietetic. He shows by laboratory experiment that the food material having the greatest potentiality of acid production is starch in a finely divided state, such as appears in pastry, bread, biscuit and chocolate, and, on the other hand, he says that "the most sapid substances are responsible for the least acid (cf. apple, potato, parsnip, orange, salad, and cane-sugar), bread crust also, by reason of the increased mastication, and therefore salivation has only developed about one-half the acid that the soft white bread has." Emphasis is laid upon food having flavor that excites salivation and for that reason acid fruits are commended. The saliva following such food especially if well masticated is alkaline and contains much ptyalin. Foods are divided into those having an alkaline or neutral reaction and those that are acid, and the author assigns to the former deleterious influences on teeth, which can be antidoted by using afterward some of the latter (acid) kind of food. So important does he regard the effects of acids that he prescribes that "all meals should contain a fair proportion of salivary excitants, and, more important still, should both commence and end with some articles of diet having an acid reaction."

We are reminded by this of Prof. Gies's suggestion, made about two years ago, that weak acids—such as dilute vinegar— be used, to rinse the mouth before or during the brushing of the teeth, to coagulate and break up the adhesion of the mucin and making easy the removal of the bacterial plaque.

The work is worthy of careful study by every practitioner, and the conclusions of the author are worthy of more thorough testing than have apparently been applied to them. Indeed it is somewhat surprising that systematic and extensive proofs of the truth of the assertions made are not offered by either of the authors, for it would seem most natural that they would have desired to make such demonstration of the reliability of their conclusions. Especially does this seem incumbent in the case of Dr. Pickerill's work, for he urges the necessity of educating the medical and the dental professions and the laity in the truths which he has demonstrated scientifically in the laboratory, and further advocates the enactment of legislation to limit the amount of starchy and sugary foods by excise taxation, just as alcoholic beverages are dealt with, and that the subject should be made a branch of the public health service.

But it seems improbable that such legislation should result from knowledge of laboratory experiments without practical demonstration of the correctness of conclusions. Juvenile Charitable Institutions furnish such a practical field for testing the results of such dietetic rules as Dr. Pickerill claims to be effective in prevention, that the question arises whether it would not be worth while to make such tests here in this country. The co-operation of the officials and of dentists interested in such institutions could no doubt easily be obtained. A study of the two works under consideration excites a desire to recommend such dietetic tests to private patients and to all who can be influenced, but it is easy to see that statements of results of any number of such isolated experiments would be far from having the convincing character that would come from tests on large numbers under control, after preliminary examinations, to be followed later by demonstrations of results.

Recognition of the value of Dr. Pickerill's work and of the importance of his conviction that he has found the efficient means of preventing decay, is far from suggesting any relaxation of the study of decay and its prevention here. It should only increase our desire to push forward such work more energetically.

The urgent need of preventing dental decay has never been presented with so much force as since the establishment of free dental clinics.

Efforts to tabulate the work of such clinics by the writer and arrive at an average cost of treatment per patient have not been quite satisfactory as yet, but we may get a suggestion of the magnitude of the problem of dental decay from the published estimate furnished the United States Bureau of Education by Dr. Thomas D. Wood, Professor of Physical Education in Teachers' College of Columbia University. He is reported to have stated that of the twenty million school children in this country, ten million have defective teeth, which are interfering with their health! What could all the dentists of this country do toward repairing the teeth of ten million children?

Compare for a moment governmental interest in the subject of the chestnut tree blight. The national Government has made an appropriation to assist in antagonizing the blight, and some of the Middle and Eastern States have also made appropriations for the same purpose that require six figures for their expression. The dental blight on the children is not so easily observed, but the importance and necessity of preventing its continuance is being constantly more appreciated.

We cannot at once accept the conclusions of Dr. Wallace and of Dr. Pickerill that the problem is solved. We look for proofs and meantime urge the necessity of more study and of making known the need of money to carry on the investigations necessary to a better understanding of the subject. It is evident that interest has been growing in foreign countries. It is hardly supposable that this country will lag behind in such work.—*Journal of Allied Societies.*

